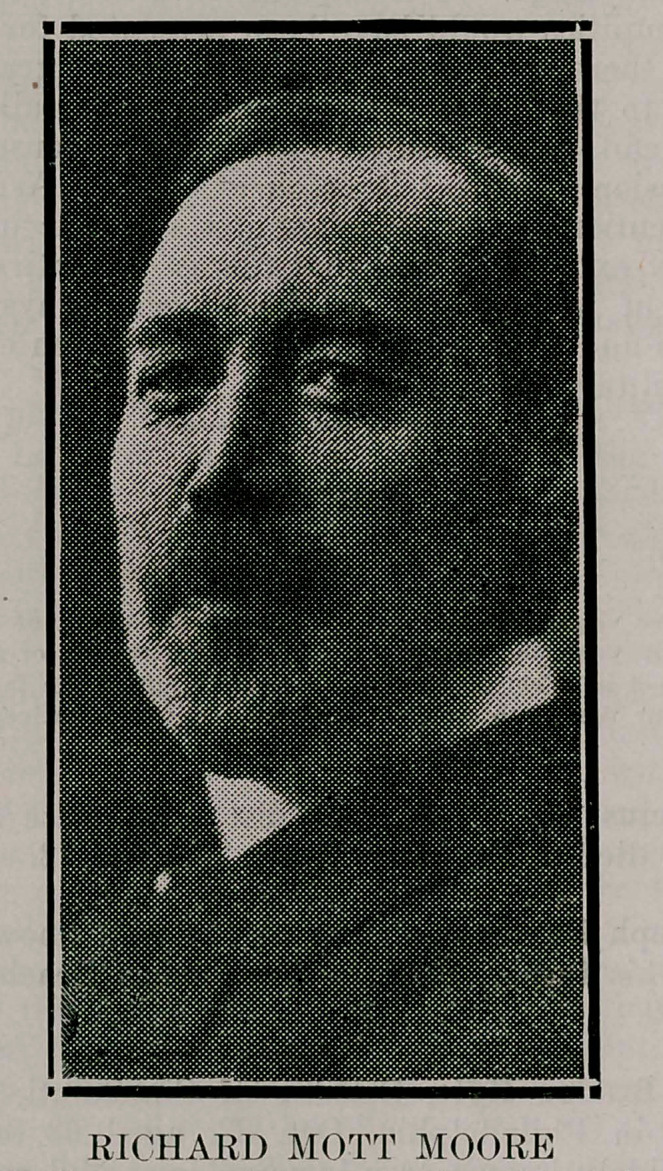# Richard Mott Moore

**Published:** 1916-11

**Authors:** W. B. Jones, Joseph W. McGill, Charles E. Darrow, Charles D. Young, E. W. Mulligan, George W. Goler


**Richard Mott Moore** died Sept. 13, 1916, at his home in Rochester, N. Y., after a long illness. He was one of the very foremost physicians of Rochester, nearly equal in renown to his illustrious father, Edward Mott Moore, a man widely known and esteemed throughout America.

He was born in Rochester on Nov. 23, 1855. His mother was Lucy Prescott a native of Vermont. He was educated in private schools in Rochester and in the University of Rochester. After two years he left however to take up his medical training in the University of Buffalo at which school his father was Professor of Surgery, though living and practicing in Rochester. He travelled after the completion of his course and attended Albany Medical School for a time. He too became an instructor in the University of Buffalo for a time.

For thirty-five years he practiced in Rochester and for many years was a leader in professional thought and activity.

RICHARD MOTT MOORE

He became highly esteemed and greatly beloved by his professional brethren, as well as by all those with whom he came into contact. He was one of the founders of the Academy of Medicine and served as president for several terms. He was for many years a Censor of the County Medical Society and a member of the Milk Commission. He was for some time a member of the Board of Health of Rochester.

Dr. Moore was much interested in general science. He was a member of the Rochester Academy of Science. He was an entomologist of some note being particularly interested in the collection of beetles.

He is survived by his wife, one daughter and three brothers, Dr. Edward Mott Moore, 2nd, Samuel P. Moore, both of Rochester, and Frederick P. Moore of Pittsburgh.

On the second day following his death there was held a joint meeting of all the Medical Societies of Rochester, and the following resolutions were adopted:

Whereas, Richard Mott Moore, for more than thirty-five years an active practitioner of medicine in the City of Rochester, and for nearly the whole of this time a member in the established medical societies or a charter member of those founded during his active life, has been removed by death, and

Whereas, The Monroe County Medical Society, the Academy of Medicine, the Rochester Medical Association, the Pathological Society and the Hospital of Medical Society are now assembled in special meeting to voice their appreciation of the life and work of Richard Mott Moore, both as a man and a practitioner of medicine.

Now, therefore, we, the members of these societies here assembled, believe that we have lost in Dr. Moore a man of character, ability, integrity and lofty purpose. As a physician among physicians we wish to testify to his helpfulness in council, as well as his ready and earnest enlistment in every good cause for the advancement of high professional character. As a physician to patients we give evidence of what we knew him to be at the bedside. Simplicity, patience, kindliness, perseverance, unsparing energy were the dominant traits of his character in all his dealings with patients, and these he inspired with something of his courage. Few among us can hope to carry to our patients the calm and comfort and the solace which his presence brought to those to whom he ministered. The kindliness, the cheer, the comfort to rich and poor alike, made him sought after by a large clientele.

Through all the years of his early and more robust manhood he gave to his patients all that was in him; and later in life, when pain and long suffering kept him sleepless through many weary nights of vigil, he still gave to his patients, both in the home and in the hospital, the same service as in his years of full vigor. Few ever heard him complain. His own ills he kept to himself. The ills of others, their pain, and the measures for its relief were always nearest his heart. Even in his last sickness he spoke little of himself. If he had pain he was like the sturdy Samuel Johnson, when he said, ''And if I have pain, I trust I shall bear it as a man.''

''So, as a physician to physicians, as a physician to patients, as well as in civic affairs, to which he gave no small measure of his busy life, he met reverses, rebuffs, criticisms, compliments and successes, with the same engaging smile and gentle demeanor. He had success in practice far above that which is given to most men, and not until his last sickness did anyone, however poor, go away unattended.

So, as physician, scientist, friend, brother, husband, father, he fulfilled a high purpose in the world. Now he is gone and we, his sorrowing survivors, ask that these resolutions be spread upon the minutes of our societies and conveyed to his family as a small tribute to one whose memory we shall cherish as long as we endure in life.

W. B. JONES,
JOSEPH W. McGILL,
CHARLES E. DARROW,
CHARLES D. YOUNG,
E. W. MULLIGAN,
GEORGE W. GOLER.

## Figures and Tables

**Figure f1:**